# (Dys)regulation of Protein Metabolism in Skeletal Muscle of Humans With Obesity

**DOI:** 10.3389/fphys.2022.843087

**Published:** 2022-03-08

**Authors:** Eduardo D. S. Freitas, Christos S. Katsanos

**Affiliations:** ^1^School of Life Sciences, Arizona State University, Tempe, AZ, United States; ^2^Department of Physiology and Biomedical Engineering, Mayo Clinic in Arizona, Scottsdale, AZ, United States

**Keywords:** obesity, muscle, proteome, metabolic disease, myopathology

## Abstract

Studies investigating the proteome of skeletal muscle present clear evidence that protein metabolism is altered in muscle of humans with obesity. Moreover, muscle quality (i.e., strength per unit of muscle mass) appears lower in humans with obesity. However, relevant evidence to date describing the protein turnover, a process that determines content and quality of protein, in muscle of humans with obesity is quite inconsistent. This is due, at least in part, to heterogeneity in protein turnover in skeletal muscle of humans with obesity. Although not always evident at the mixed-muscle protein level, the rate of synthesis is generally lower in myofibrillar and mitochondrial proteins in muscle of humans with obesity. Moreover, alterations in the synthesis of protein in muscle of humans with obesity are manifested more readily under conditions that stimulate protein synthesis in muscle, including the fed state, increased plasma amino acid availability to muscle, and exercise. Current evidence supports various biological mechanisms explaining impairments in protein synthesis in muscle of humans with obesity, but this evidence is rather limited and needs to be reproduced under more defined experimental conditions. Expanding our current knowledge with direct measurements of protein breakdown in muscle, and more importantly of protein turnover on a protein by protein basis, will enhance our understanding of how obesity modifies the proteome (content and quality) in muscle of humans with obesity.

## Introduction

Obesity is a widespread and fast growing health concern (Hales et al., 2020). Thus, comorbidities (i.e., diabetes and heart disease) occurring as consequence of obesity carry relevant social impact and economic burden. Obesity affects function of tissues across the body, including the skeletal muscle, and current evidence shows poor quality of muscle in humans with obesity (Valenzuela et al., 2020). Muscle quality is evaluated as strength output per unit of muscle mass and has greater implications for physical function and performance of activities of daily living when compared to absolute muscle mass. Interestingly, muscle quality is reduced even in muscles of lower extremities, despite these muscles being exposed to a “training effect” due to increased body weight in obesity (Tomlinson et al., 2016). Effects of obesity on muscle mass and function become generally more evident at older age, at which time are revealed in the clinical setting as “sarcopenic obesity” (Stenholm et al., 2008).

Altered muscle fiber phenotype is a hallmark of morphological changes observed in skeletal muscle of humans with obesity (Wade et al., 1990; Hickey et al., 1995; Tanner et al., 2002; Stuart et al., 2013). This is associated with altered content in the isoforms of the protein myosin heavy chain, as well as overall distortions in the proteome of skeletal muscle in humans with obesity (Hwang et al., 2010). As such, protein metabolism in skeletal muscle of humans with obesity induces a discrete muscle phenotype that is of particular importance for overall/whole-body metabolism. Specifically, and because it is the largest insulin-sensitive tissue in the body, skeletal muscle has the primary role for whole-body glucose utilization (Stump et al., 2006), a process that is, however, impaired in humans with obesity and in the presence of distorted muscle proteome (Hwang et al., 2010). Furthermore, evidence accumulated over the last few years shows that disruptions in cellular proteome homeostasis accelerates the effects of aging (Basisty et al., 2018; Santra et al., 2019).

Until recently, there was very little evidence on protein metabolism in skeletal muscle of humans with obesity, despite original evidence collected in rodents decades ago showing that obesity is linked to adverse effects on protein metabolism in muscle (Durschlag and Layman, 1983; Lanza-Jacoby and Kaplan, 1984; Friedman et al., 1990; Herrero et al., 1997). Early efforts to understand protein metabolism in humans with obesity focused on whole-body protein turnover, but subsequent studies provided original evidence for impaired protein turnover occurring specifically in muscle of these individuals (Guillet et al., 2011; Katsanos and Mandarino, 2011). Over the last ~10 years, various studies have generated considerable amount of evidence describing the effects of obesity on protein content and turnover in skeletal muscle. Effects on skeletal muscle protein turnover are reported at the levels of whole-/mixed-muscle protein, functionally related groups of proteins, or individual proteins within muscle. Findings from these studies do not always support the same conclusions, prolonging the controversy about the effects of obesity on muscle protein metabolism. It is important to note that the role of plasma insulin on muscle protein metabolism (Abdulla et al., 2016) and the associated insulin resistance in obesity, the effects of fasting on muscle protein metabolism (Bak et al., 2016), and whether protein metabolism is assessed under a fasting or fed state, as well as the interactions between feeding and physical activity on muscle protein metabolism (Miller, 2007), are all important factors that need to be considered when interpreting data from studies investigating the regulation of muscle protein metabolism in obesity.

Goals of this review are to summarize the body of evidence describing the protein metabolism in muscle of humans with obesity, provide possible insights into discrepant findings, describe mechanisms implicated in altered protein metabolism in muscle of these individuals, and emphasize areas in need of more research. By discussing a framework to guide this research, we expect to achieve a more complete understanding of how obesity affects protein metabolism in skeletal muscle of humans. We have placed particular focus on studies conducted with young and middle-aged humans to avoid the concurrent effects of aging on impairing protein metabolism in muscle of older adults with obesity.

## Fasted-State Mixed-Muscle Protein Synthesis and Breakdown

Total protein in skeletal muscle is under constant turnover determined by the processes of breakdown of old and/or damaged proteins and synthesis of new ones. Together, the biological processes of protein synthesis and breakdown maintain protein mass in skeletal muscle, and the rate of synthesis is of particular physiological importance because it also determines the rate of renewal of the proteins in muscle. That is, the rate by which malfunctional, damaged, and/or old proteins in muscle are replaced with newly synthesized ones, thus maintaining proper mechanical and metabolic function in skeletal muscle. Table 1 summarizes the evidence from available studies that have evaluated synthesis and breakdown of muscle protein in skeletal muscle of humans with obesity and relative to those of non-obese humans.

**Table 1:** Summary of studies evaluating protein synthesis and/or breakdown in skeletal muscle of humans with obesity in the fasted state.

Study	Status (Sex)	Age	Outcome measures	Results
Bak et al., 2016	OB (M = 9, F = 0)	24	Forearm phenylalanine R_a_	OB < LN
	LN (M = 9, F = 0)	24	Forearm phenylalanine R_d_	OB < LN
Beals et al., 2016	OB (M = 5, F = 5)	27	Myofibrillar protein FSR	OB ~ LN
	LN (M = 5, F = 5)	24		
Beals et al., 2017	OB (M = 5, F = 5)	27	Mitochondrial protein FSR	OB ~ LN
	LN (M = 5, F = 5)	24		
Beals et al., 2018	OB (M = 5, F = 4)	22	Myofibrillar protein FSR	OB ~ LN
	LN (M = 4, F = 5)	21	Sarcoplasmic protein FSR	OB ~ LN
Chevalier et al., 2015	OB (M = 11, F = 0)	44	Myofibrillar protein FSR	OB ~ LN
	LN (M = 8, F = 0)	21	Sarcoplasmic protein FSR	OB ~ LN
Guillet et al., 2009	OB (M = 6, F = 0)	24	Mixed-muscle protein FSR	OB < LN
	LN (M = 8, F = 0)	26	Mitochondrial protein FSR	OB < LN
Hulston et al., 2018	OB (M = 7, F = 1)	24	Mixed-muscle protein FSR	OB ~ LN
	LN (M = 8, F = 1)	27		
Kouw et al., 2019	OB (M = 12, F = 0)	48	Myofibrillar protein FSR	OB ~ LN
	LN (M = 12, F = 0)	43		
Murton et al., 2015	OB (M = 11, F = 0)	66	Myofibrillar protein FSR	OB ~ LN
	LN (M = 15, F = 0)	66		
Patterson et al., 2002	OB (M = 0, F = 5)	37	Forearm leucine Ra	OB < LN
	LN (M = 0, F = 5)	29		
Serrano et al., 2021	OB (M = 3, F = 3)	30	Mixed-muscle protein FSR	OB ~ LN
	LN (M = 3, F = 4)	29	Mitochondrial protein FSR	OB ~ LN
Tran et al., 2016	OB (M = 4, F = 3)	39	Mixed-muscle protein FSR	OB < LN
	LN (M = 3, F = 3)	33		
Tran et al., 2018	OB (M = 6, F = 4)	36	Mixed-muscle protein FSR	OB < LN
	LN (M = 4, F = 6)	35	Mitochondrial protein FSR	OB < LN
Tran et al., 2019	OB (N/A)	37	Mixed-muscle FSR	OB < LN
	LN (N/A)	37		

OB, obese; LN, lean/non-obese; M, males; F, females; FSR, fractional synthesis rate of protein; R_d_, rate of muscle amino acid disappearance (i.e., protein synthesis); R_a_, rate of amino acid muscle appearance (i.e., protein breakdown); ˜, comparable/not significantly different. N/A, not available.

Studies assessing the effects of human obesity on the metabolism of mixed-muscle (i.e., overall) protein differ in their conclusions. To illustrate, Guillet et al. (2009) were one of the first showing that after an overnight fast the rate of synthesis of mixed-muscle protein, measured by isotope tracers of amino acids, is lower in humans with obesity compared to controls. On the other hand, Hulston et al. (2018) reported no differences in mixed-muscle protein synthesis between humans with obesity and lean humans. However, other studies performed under comparable experimental conditions, are in agreement with the findings from Guillet et al. (2009). These studies have reported lower synthesis of mixed-muscle protein in humans with obesity by using amino acid tracers and either the precursor-product approach (Tran et al., 2016, 2018) or the arteriovenous balance approach (i.e., rate of tracer disappearance into muscle; Bak et al., 2016). Interestingly, and based on findings from the latter report (Bak et al., 2016), extended fasting up to 72 h does not reduce further the overall protein synthesis in muscle of humans with obesity when compared to a 12-h fasting (i.e., typical duration of an overnight fasting period in most studies investigating muscle protein metabolism in humans). However, such extended fasting decreases protein synthesis in non-obese subjects (Bak et al., 2016), indicating possibly an earlier effect of the absence of dietary intake on modifying the time course of the decrease in fasted-state muscle protein synthesis in obesity. Contrary to these findings, however, other lines of evidence show no differences in the synthesis of mixed-muscle protein in the fasted state between humans with obesity and lean controls (Hulston et al., 2018; Serrano et al., 2021), suggesting that obesity, *per se*, may not always affect the synthesis rate of overall protein in muscle in the fasted state.

Breakdown rate of protein within muscle is important in determining the total content of protein in muscle. However, and although lower muscle protein breakdown may increase the content of protein within a given muscle protein pool, it results in accumulation of older, possibly damaged proteins (Jaleel et al., 2010). When compared to that of protein synthesis, data describing protein breakdown in muscle of humans with obesity are rather limited. Evidence using amino acid tracers in conjunction with the arteriovenous balance approach, shows that forearm amino acid release, which is experimentally employed to describe muscle protein breakdown, is lower in women with obesity compared to lean women (Patterson et al., 2002). A separate study using a comparable experimental approach found lower muscle protein breakdown also in men with obesity when compared to lean men (Bak et al., 2016). Therefore, although limited, current evidence shows rather consistently that overall protein breakdown in muscle is lower in humans with obesity. However, and despite lower muscle protein breakdown in obesity, overall protein balance in muscle is not different between subjects with obesity and lean subjects, because the synthesis of protein is also lower in the subjects with obesity (Bak et al., 2016).

Lower rate of synthesis concurrently with lower rate of breakdown of overall protein in skeletal muscle of humans with obesity (Bak et al., 2016) suggests lower rate of renewal of the overall protein pool in muscle of these individuals. Collectively, these responses may explain the lower muscle quality observed in humans with obesity (Valenzuela et al., 2020), and secondary to the existence of older/more metabolically damaged, less functional proteins (Jaleel et al., 2010). Reductions in the processes of protein synthesis and breakdown in skeletal muscle in humans with obesity could result from mechanisms that independently regulate these processes in skeletal muscle and induced in response to the specific metabolic environment of obesity. Alternatively, lower muscle protein synthesis observed concurrently with lower muscle protein breakdown in humans with obesity (Bak et al., 2016) may imply that reduction in one of the processes mediates reduction in the other process in muscle in order to maintain a given protein content within muscle in obesity.

Because almost all evidence to date describing effects of obesity on muscle protein turnover in humans comes from studies measuring only the synthesis rate of muscle protein, we currently have incomplete understanding of the breakdown of protein in muscle in obesity, and as such of the dynamic regulation of overall protein content and quality in muscle of humans with obesity. Further limitation of current studies is that measurements performed at the mixed-muscle protein level alone mask effects of obesity on protein turnover of given proteins, considering that synthesis rates differ greatly between individual proteins within skeletal muscle (Jaleel et al., 2008).

## Fasted-State Synthesis and Breakdown of Distinct Protein Pools in Muscle

Proteomics analyses show differences in the content of total protein of functionally linked groups of proteins in skeletal muscle between subjects with obesity and non-obese controls (Hwang et al., 2010; Kras et al., 2018). Moreover, obesity alters the content of functionally linked proteins, such as the mitochondrial proteins, on an individual protein basis within muscle (Kras et al., 2018). These lines of evidence underline that, unless the content of total protein in muscle is the main outcome (i.e., accumulation of muscle mass), it is important to evaluate synthesis and breakdown of protein in muscle at the levels of functionally linked groups of proteins and/or in a protein by protein basis. Such knowledge is summarized next, but it is very limited because measuring currently breakdown of groups of proteins or individual proteins in muscle of humans is technically challenging. However, such determinations are essential in evaluating effects of obesity on either content or the rate of renewal and degradation/removal of protein(s) that have functional and metabolic implications in skeletal muscle in obesity.

### Myofibrillar Proteins

Myofibrillar proteins constitute approximately half of the total protein in skeletal muscle. In addition to their quantitative importance within the overall muscle proteome, myofibrillar proteins support myofiber structure and form the contractile apparatus of skeletal muscle. Thus, maintenance of the myofibrillar protein pool is undoubtedly critical for overall musculoskeletal health and function. Chevalier et al. (2015) found that the synthesis rate of myofibrillar protein is not different between subjects with obesity and normal-weight controls. This agrees with subsequent studies reporting effects of obesity on myofibrillar protein synthesis (Beals et al., 2016, 2018; Kouw et al., 2019). However, relevant evidence about the rate of breakdown of myofibrillar protein in the fasted state in muscle of humans with obesity is currently lacking.

### Sarcoplasmic Proteins

Sarcoplasmic proteins comprise about a third of the overall protein pool in skeletal muscle, and because of that, the sarcoplasmic protein content is quantitatively important in the context of overall protein metabolism in muscle. Although limited, current evidence consistently shows that obesity does not alter the rate of synthesis of the overall sarcoplasmic protein pool in skeletal muscle of humans (Chevalier et al., 2015; Beals et al., 2018).

### Mitochondria

Mitochondrial proteins constitute a small portion of the overall muscle proteome. However, they regulate energy metabolism, as well as other biological processes (i.e., production of reactive oxygen species), of great metabolic importance in skeletal muscle. Because such biological processes are altered in muscle of humans with obesity, with implications for developing insulin resistance and Type 2 Diabetes (Abdul-Ghani et al., 2009), regulation of mitochondrial protein metabolism in humans with obesity has received particular attention.

Some studies have found lower mitochondrial protein synthesis in muscle of subjects with obesity (Guillet et al., 2009; Tran et al., 2018). Contrary to these findings, other studies have reported no differences in muscle mitochondrial protein synthesis between humans with obesity and non-obese controls (Beals et al., 2017; Serrano et al., 2021). However, lower content of mitochondrial proteins in muscle of humans with obesity (Hwang et al., 2010; Lefort et al., 2010; Kras et al., 2018) implies that mitochondrial protein turnover is altered in obesity. This may be a consequence of lower rate of synthesis of mitochondrial proteins, and as reported in some studies (Guillet et al., 2009; Tran et al., 2018), or higher rate of breakdown of mitochondrial proteins in muscle of humans with obesity, but evidence with respect to the latter is currently lacking. It is import to note that findings discussed in the paragraph above relate specifically to the mitochondrial sub-fraction found under the sarcolemma in skeletal muscle. This is because isolation of mitochondria from the intermyofibrillar region to determine synthesis rates of this mitochondrial sub-fraction using standard precursor-product isotopically labeled tracers is technically difficult (i.e., requires the use of a digestive enzyme for the isolation of the intermyofibrillar mitochondria that dilutes the stable isotope enrichment of the protein synthesized *in vivo*). However, lower content of total and individual mitochondrial proteins isolated from the intermyofibrillar region in subjects with obesity (Chomentowski et al., 2011; Kras et al., 2018) suggests that turnover of mitochondrial proteins within the intermyofibrillar region is also affected in muscle of humans with obesity.

Evidence for impaired mitochondrial protein synthesis in skeletal muscle of humans with obesity (Guillet et al., 2009; Tran et al., 2018) is not supported by all studies (Beals et al., 2017; Serrano et al., 2021). There is possibly heterogeneity in the response of synthesis of mitochondrial protein in muscle of humans with obesity. There is clear need to understand the conditions under which mitochondrial protein synthesis and content are adversely affected in skeletal muscle of humans with obesity. This is important given that mitochondria in muscle are implicated not only in developing obesity-associated insulin resistance (Pileggi et al., 2021), but also in impairing muscle proteostasis itself and maintenance of muscle mass (Coen et al., 2018). A limitation when evaluating protein turnover at the overall mitochondrial proteome is that this approach does not provide any information about synthesis rates of either individual mitochondrial proteins or functionally linked mitochondrial protein complexes. While content of some mitochondrial protein complexes is higher, that of others is actually lower in muscle of humans with obesity (Kras et al., 2018), effects likely mediated by same-direction responses in the synthesis rate of their respective proteins. Therefore, effects of obesity on impairing the synthesis rates of specific mitochondrial proteins are not readily evident at the overall mitochondrial proteome level.

### Individual Proteins

Almost all studies investigating skeletal muscle protein turnover in obesity have focused on either mixed-muscle protein or protein pools restricted to certain cellular compartments that include the myofibrillar, sarcoplasmic, or mitochondrial proteins. However, individual proteins in muscle display quite wide variation in their synthesis rates (Jaleel et al., 2008; Camera et al., 2017), and obesity may alter the synthesis rate of proteins in skeletal muscle on a protein by protein basis. In this regard, although measuring rates of synthesis and breakdown on a protein by protein basis *in vivo* using stable isotope tracers are technically challenging, it is of great importance in deepening our understanding of protein turnover in muscle of humans with obesity. In addition to describing mechanisms (i.e., synthesis vs. breakdown) that determine the content of proteins in muscle, understanding of protein turnover on a protein by protein basis is more informative with respect to linking protein metabolism to function in skeletal muscle.

Limited evidence to date on protein turnover at the level of individual proteins shows that subjects with obesity have lower synthesis rate of the beta subunit of the ATP synthase (*β*-F1-ATPase; Tran et al., 2019), a protein of the mitochondrial Complex V that serves as the catalytic site for ATP production. Lower synthesis rate of *β*-F1-ATPase may explain lower abundance of this protein in muscle of humans with obesity (Hojlund et al., 2010; Hwang et al., 2010; Tran et al., 2016). Notably, expression of the *β*-F1-ATPase in muscle of humans with obesity is not affected at the transcription level (Tran et al., 2019), suggesting impaired translation of this protein in muscle in obesity. Moreover, lower synthesis rate of *β*-F1-ATPase in muscle of humans with obesity is observed concomitant with impaired function of the ATP synthase (Tran et al., 2019) and lower concentration of ATP in muscle of humans with obesity (Lennmarken et al., 1986). Collectively, these lines of evidence link impaired synthesis rate of a protein to impaired function in skeletal muscle in obesity.

Unfortunately, there is a dearth of information about protein turnover on a protein by protein basis in muscle of humans with obesity. Rates of synthesis and breakdown are affected differently among the numerous individual proteins in skeletal muscle in response to consumption of a high-fat diet, which, to some extent, may reflect a metabolic state resembling obesity (Camera et al., 2017). Describing turnover rate of individual proteins is key to enhance our understanding of the mechanisms that determine the proteome and metabolic function in muscle of humans with obesity. In this regard, the specific functional and metabolic characteristics of muscle in obesity result largely from differences observed in muscle fiber phenotype in these individuals (Wade et al., 1990; Hickey et al., 1995; Tanner et al., 2002; Stuart et al., 2013) and which are secondary to differences in the expression of the isoforms of the myosin heavy chain protein in muscle (Campbell et al., 2016).

## Regulation of Muscle Protein Turnover by Acute Exercise

It is well established that acute exercise stimulates both protein synthesis (reviewed in Miller, 2007; Atherton and Smith, 2012) and protein breakdown in skeletal muscle. However, the increase in muscle protein breakdown after exercise is comparatively lower than that of protein synthesis (Tipton et al., 2018), in a way that exercise results in an overall accretion of protein in muscle. Thus, increase in protein synthesis by exercise not only remodels/renews the muscle proteome, but it also provides for increase in the overall protein in skeletal muscle. In non-obese humans, both resistance and aerobic exercise stimulate protein synthesis in muscle, with resistance exercise favoring the synthesis of myofibrillar protein and aerobic exercise favoring the synthesis of mitochondrial protein (Wilkinson et al., 2008).

Evidence first obtained in animal models of obesity shows that obesity impairs protein metabolism in skeletal muscle in response to exercise. Specifically, diet-induced obesity in mice attenuates the growth of skeletal muscle in response to mechanical overload (Sitnick et al., 2009). Also, Zucker rats with obesity show impaired mitochondrial biogenesis in response to resistance exercise (Nilsson et al., 2010). Recent evidence using rodent models of obesity shows that impaired response in protein synthesis in muscle to resistance exercise occurs specifically in Type I and IIa muscle fibers (Ato et al., 2021). Understanding on whether impairments in muscle protein metabolism observed in rodent models of obesity occurs also in humans with obesity has only recently gained attention. To date, only few studies relative to the studies in the overall field of muscle protein metabolism in humans have investigated protein turnover in muscle of humans with obesity in response to resistance and/or aerobic exercise. The findings from these studies are summarized in Table 2.

**Table 2:** Summary of studies evaluating protein synthesis in skeletal muscle of humans with obesity after acute exercise.

Study	Status (Sex)	Age	Exercise protocol	Outcome measures	Results
^#^ Beals et al., 2018	OB (M = 5, F = 4)	22	Resistance: unilateral leg extension (4 × 10–12 reps at 65–70% of 1 RM)	Myofibrillar protein FSR	↑ OB < ↑ LN
	LN (M = 4, F = 5)	21		Sarcoplasmic protein FSR	↑ OB ~ ↑ LN
Hulston et al., 2018	OB (M = 7, F = 1)	24	Resistance: unilateral knee-extension (1 × 10 reps at 30% of 1 RM and 4 × 10 reps at 70% of 1 RM)	Mixed-muscle protein FSR	↑ OB ~ ↑ LN
	LN (M = 8, F = 1)	27			
Serrano et al., 2021	OB (M = 3, F = 3)	30	Aerobic: 45 min cycling (65% of heart rate reserve)	Mixed-muscle protein FSR	↔ OB ~ ↔ LN
	LN (M = 3, F = 4)	29		Mitochondrial protein FSR	↔ OB ~ ↔ LN
Smiles et al., 2019	OW/OB (M = 10, F = 0)	40	Aerobic: 30 min cycling (60% VO_2peak_) + Resistance: unilateral knee-extension (7 × 5 + 1 set to volitional fatigue at 80% of 1 RM) + lipid infusion	Mixed-muscle protein FSR	↑

OB, obese; OW, overweight; LN, lean/non-obese; M, males; F, females; FSR, fractional synthesis rate of protein; VO_2peak_, peak oxygen uptake; 1RM, one-repetition maximum; ↔, *no significant change from fasted state;* ↑, *significant increase from fasted state,* ˜, comparable/not significantly different.

# In the presence of fed state.

With respect to resistance exercise, evidence obtained more than two decades ago, shows that increased adiposity impairs strength adaptations in muscle of young individuals performing resistance exercise training (Falk et al., 2002). More recent evidence at the molecular level shows that the increase in mixed-muscle protein synthesis following resistance exercise [i.e., four sets of knee-extension to volitional fatigue at 70% of one-repetition maximum (1-RM)] in humans with obesity is comparable to that observed in lean humans (Hulston et al., 2018). However, under a rather comparable resistance exercise protocol (i.e., 4 × 10–12 repetitions of knee-extension at 65% of 1-RM), the post-exercise increase in myofibrillar protein synthesis was lower in humans with obesity (Beals et al., 2018). On the other hand, in the latter report, the corresponding synthesis rate response within the sarcoplasmic protein pool was not different from that in lean subjects (Beals et al., 2018). Collectively, these lines of evidence show that impaired protein synthesis in muscle of humans with obesity in response to resistance exercise occurs in certain protein pools that include the contractile proteins.

Concerning acute aerobic exercise, only few studies have investigated the effects of this type of exercise modality on protein metabolism in muscle of humans with obesity. In lean subjects, acute aerobic exercise performed in a fasted state appears to provide adequate stimulus to upregulate synthesis of mixed-muscle (Carraro et al., 1990; Harber et al., 2010) as well as myofibrillar (Di Donato et al., 2014) proteins. However, there is also evidence indicating that acute aerobic exercise does not increase either mixed-muscle (Serrano et al., 2021) or mitochondrial (Di Donato et al., 2014; Serrano et al., 2021) protein synthesis in lean subjects. It is possible that this discrepancy in the findings between studies is the result of the specific experimental protocols employed in each study. Therefore, it is critical to use experimental approaches (i.e., type of acute exercise and timing of protein turnover measurements) that show upregulation in protein synthesis in muscle of non-obese subjects if defects in protein synthesis in muscle of subjects with obesity are to be detected. Important consideration when studying the effects of acute aerobic exercise on muscle protein synthesis is that this type of exercise decreases the concentration of amino acids in plasma (Struder et al., 1999; Serrano et al., 2021). Decrease in the concentration of amino acids in plasma at levels below those found in the fasted state inhibits protein synthesis in skeletal muscle (Kobayashi et al., 2003). On the other hand, aerobic exercise performed in association with a fed state increases mitochondrial protein synthesis (Wilkinson et al., 2008; Donges et al., 2012), which underlines the important role of increased amino acids in plasma in stimulating protein synthesis in muscle in response to aerobic exercise. Therefore, effects of obesity on impairing the response of protein synthesis in muscle to acute aerobic exercise are likely to become evident under experimental protocols that increase, or at least prevent decrease, in the concentration of plasma amino acids post-exercise, but such evidence is currently lacking.

Although not necessarily an identical metabolic state, increasing the concentration of free fatty acids in plasma induces a metabolic environment that resembles that of obesity (Boden, 2008). This metabolic environment impairs glucose metabolism in muscle of lean humans, a metabolic effect that is comparable to that observed in muscle of humans with obesity. Likewise, increasing free fatty acids in plasma of lean humans impairs synthesis of mixed-muscle protein induced by increase in plasma amino acids (Stephens et al., 2015). However, acute aerobic exercise overcomes the negative effects of increased plasma free fatty acids on protein synthesis (Smiles et al., 2019), suggesting that acute aerobic exercise may counteract impairments on muscle protein metabolism induced by lipotoxicity, a metabolic state present in obesity (Ye et al., 2019). However, there is a critical need for studies to address this gap in the literature regarding the effects of obesity-associated lipotoxicity in modifying the response of protein synthesis to acute aerobic exercise. Such evidence is particularly important with respect to specific protein pools as well as individual proteins within skeletal muscle that have metabolic and functional implications for health.

In summary, current evidence shows that obesity impairs synthesis in the myofibrillar protein pool in response to acute resistance exercise. It is important to note that defects in protein synthesis in muscle of humans with obesity after exercise are evident in association with increased availability of nutrients in plasma, making it difficult to attribute observed defects in protein synthesis in muscle in obesity specifically to impaired response to the exercise stimulus, *per se*. Along such experimental limitations, it is also worth noting that humans with obesity are generally physically inactive (Petersen et al., 2004) and that physical inactivity exacerbates anabolic resistance to protein synthesis in skeletal muscle (Smeuninx et al., 2017). Moreover, protein synthesis in muscle in response to resistance exercise in subjects with obesity is lower than that of lean controls when both subject groups are reported to be inactive (Beals et al., 2018), but it is not different between groups when both groups are physically active (Hulston et al., 2018). Therefore, properly designed studies are key to determine whether obesity directly impairs protein metabolism in response to acute exercise in muscle of humans.

## Regulation by Fed State/Amino Acids

Feeding induces overall protein accrual in skeletal muscle by increasing the rate of protein synthesis and concomitantly decreasing, although to lower extent, that of protein breakdown in muscle (Burd et al., 2009). Among nutrients, amino acids are the most powerful stimuli for increasing protein synthesis in muscle (Wolfe, 2002). Therefore, several investigators have turned their attention to the effects of obesity on muscle protein synthesis in the fed state, namely, following protein ingestion or intravenous supply of amino acids. These findings to date are summarized in Table 3. Overall, some studies reveal comparable responses in protein synthesis in muscle to increase in plasma amino acids between humans with obesity and lean controls, whereas other studies show that this response is impaired in humans with obesity. Some evidence shows that obesity impairs protein synthesis in muscle in the fed state (Beals et al., 2016), whereas other evidence shows that the response of protein synthesis in muscle of humans with obesity is comparable to that of lean humans both in the fed state (Kouw et al., 2019) and under conditions that resemble a fed state (Chevalier et al., 2015; Tran et al., 2018). In fact, hyperaminoacidemia induces acute increase in protein synthesis in muscle of humans with obesity that is quantitatively greater (Tran et al., 2018), although not always significant (Chevalier et al., 2015), than that in lean subjects.

**Table 3:** Summary of studies evaluating protein synthesis and/or breakdown in skeletal muscle of humans with obesity in the fed state.

Study	Status (Sex)	Age	Outcome measures	Results
Beals et al., 2016	OB (M = 5, F = 5)	27	Myofibrillar protein FSR	↔ OB < ↑ LN
	LN (M = 5, F = 5)	24		
Beals et al., 2017	OB (M = 5, F = 5)	27	Mitochondrial protein FSR	↑ OB ~ ↑ LN
	LN (M = 5, F = 5)	24		
^#^ Beals et al., 2018	OB (M = 5, F = 4)	22	Myofibrillar protein FSR	↑ OB ~ ↑ LN
	LN (M = 4, F = 5)	21	Sarcoplasmic protein FSR	↑ OB ~ ↑ LN
Chevalier et al., 2015	OB (M = 11, F = 0)	44	Myofibrillar protein FSR	↑ OB ~ ↑ LN
	LN (M = 8, F = 0)	21	Sarcoplasmic protein FSR	↑ OB ~ ↑ LN
Guillet et al., 2009	OB (M = 6, F = 0)	24	Mixed-muscle protein FSR	↑ OB < ↑ LN
	LN (M = 8, F = 0)	26	Mitochondrial protein FSR	↔ OB < ↑ LN
Kouw et al., 2019	OB (M = 12, F = 0)	48	Myofibrillar protein FSR	↑ OB ~ ↑ LN
	LN (M = 12, F = 0)	43		
Murton et al., 2015	OB (M = 11, F = 0)	66	Myofibrillar protein FSR	↔ OB < ↑ LN
	LN (M = 15, F = 0)	66	Leg phenylalanine R_a_	↓ OB < ↔ LN
Tran et al., 2018	OB (M = 6, F = 4)	36	Mixed-muscle protein FSR	↑ OB > ↑ LN
	LN (M = 4, F = 6)	35	Mitochondrial protein FSR	↑ OB ~ ↑ LN

OB, obese; LN, lean/non-obese; M, males; F, females; FSR, fractional synthesis rate of protein; R_a_, rate of muscle amino acid appearance (i.e., protein breakdown); ↔, *no significant change from fasted state;* ↑, *significant increase from fasted state;* ↓, *significant decrease from fasted state;* ~, comparable/not significantly different.

# In the presence of an exercise stimulus.

Investigations focused on given protein pools within skeletal muscle have concluded that the response of myofibrillar protein synthesis is not different between humans with obesity and lean humans under circumstances associated with a fed state (Chevalier et al., 2015). However, others have reported lower response in myofibrillar protein synthesis in humans with obesity under comparable conditions (Beals et al., 2016). Also, the response of mitochondrial protein synthesis in humans with obesity under conditions that stimulate protein synthesis in muscle (i.e., provision of nutrients, including amino acids) is reported as both impaired (Guillet et al., 2009) and responsive (Beals et al., 2017; Tran et al., 2018) when compared to that in lean humans. Such discrepant findings between studies investigating the effects of obesity on the response of protein synthesis in a given muscle protein pool may result from the unique experimental conditions employed in each study, as well as on whether the fed state is induced by meal ingestion (Beals et al., 2016) as opposed to experimental infusion of nutrients/hormones in order to mechanistically enhance the experimental design (Chevalier et al., 2015). However, it is also possible that discrepant findings in the response of muscle protein synthesis in obesity are the result of a metabolic heterogeneity associated with the (patho)physiological state of obesity. There is indeed heterogeneity in metabolic responses in humans with obesity, and not every individual with obesity is characterized by metabolic impairments (Kloting et al., 2010; Ahima and Lazar, 2013). It is reasonable to speculate that this is also true about protein metabolism in skeletal muscle of humans with obesity, and in a way that it contributes to the differences in the responses in muscle protein synthesis among the subject populations with obesity examined in the aforementioned studies.

It is technically challenging using current methodologies to directly measure protein breakdown in muscle under the non-steady state plasma amino acid concentrations of a fed state. A study employing experimental conditions that resemble a postprandial state, shows that subjects with obesity, albeit older adults, have lower rate of leg, and presumably muscle, protein breakdown during concurrent hyperinsulinemia, hyperaminoacidemia, and euglycemia (Murton et al., 2015). Although limited, this evidence suggests that muscle protein breakdown is suppressed to a greater extent in humans with obesity than non-obese humans in a fed state. Lower protein breakdown in muscle under these conditions, and in the presence of comparable response in protein breakdown in the fasted stated (Murton et al., 2015), indicates that the overall rate of renewal of the muscle proteome is reduced in humans with obesity. This response is expected to result in accumulation of less functional proteins in muscle of humans with obesity (and as discussed in the sections above), and which is in line with lower metabolic quality of muscle observed in these subjects (Murton et al., 2015).

In summary, evaluating protein turnover in a non-steady state, as in the postprandial state, is technically complex. Therefore, findings to date likely reflect the specific experimental conditions employed in each study. All these limit our ability to draw conclusions about protein metabolism in the fed state that are applicable to the general population with obesity. Factors such as meal size, timing of meal ingestion, composition of the meal, duration of metabolic measurements, along with concurrent changes in plasma hormones that may differ among humans with obesity, mediate different responses and, thus, are key factors to consider when evaluating protein turnover in the fed state in muscle of humans with obesity.

## Potential Mechanisms Impairing Protein Synthesis in Muscle of Humans With Obesity

### Intracellular Signaling

The importance of intracellular signaling for protein synthesis in muscle is most evident under the fed state, which induces acute stimulation of the overall translational machinery in muscle (Liu et al., 2002). This process is initiated by muscle signaling pathways that respond to amino acids and insulin, and signaling through the mammalian target of rapamycin (mTOR), which has central role in this process (Balage et al., 2001). Hence, disruption of mTOR signaling can be potential factor contributing to dysregulated protein metabolism in skeletal muscle of humans, including those with obesity. However, studies to date have consistently demonstrated lack of impaired mTOR signaling, and its downstream target ribosomal protein S6 kinase-1 (S6K1), in humans with obesity in the fasted state (Bak et al., 2016; Beals et al., 2016; Tran et al., 2018). In fact, activation of mTOR in the fasted-state is higher in muscle of humans with obesity (Beals et al., 2016; Tran et al., 2018). Increased mTOR activation in skeletal muscle of humans with obesity in the fasted state may result from effects of an obesity-associated insulin resistance on increasing the plasma amino acids (Felig et al., 1969; Adams, 2011), and given the role of increased concentrations of plasma amino acids (and insulin) in activating mTOR (Tremblay et al., 2005). In agreement with this evidence obtained in studies investigating protein metabolism, the metabolic state of obesity is often linked to prolonged activation of mTOR, a condition that induces tissue insulin resistance (Tremblay and Marette, 2001; Khamzina et al., 2005). Moreover, during nutrient oversupply, phosphorylation of mTOR remains elevated, while that of S6K1 increases more in subjects with obesity compared to lean subjects (Beals et al., 2016; Tran et al., 2018). These findings argue against impaired activation of mTOR or mTOR to S6K1 signaling in impairing protein synthesis in skeletal muscle of humans with obesity, either in the fasted state (Bak et al., 2016; Tran et al., 2016) or in the presence of nutrients that stimulate protein synthesis in muscle (Beals et al., 2016).

In regards to protein breakdown, there is currently lack of evidence about the mechanisms that may mediate the observed lower rate of protein breakdown in muscle of humans with obesity (Patterson et al., 2002; Bak et al., 2016). Current evidence suggests decreased expression of autophagy-related genes in skeletal muscle from severely insulin-resistant patients with Type 2 Diabetes (Moller et al., 2017), and these same mechanisms may play role in attenuating muscle protein breakdown in obesity, and where insulin resistance is a common manifestation. Because activation of mTOR inhibits autophagy in skeletal muscle (Castets et al., 2013), obesity-induced chronic activation of mTOR may explain the lower protein breakdown observed in muscle of humans with obesity (Patterson et al., 2002; Bak et al., 2016). Increased concentrations of plasma insulin in the basal state in humans with insulin resistance suppresses skeletal muscle protein breakdown (Denne et al., 1995). However, currently, we cannot conclusively attribute a role of mTOR activation by insulin on reducing protein breakdown in muscle of humans with obesity. This is because physiological insulin concentrations, although decrease markers of autophagy in muscle, this effect does not necessarily involve activation of the mTOR-S6K1 signaling pathway (Kruse et al., 2015).

Although acute activation of mTOR is physiologically important to induce protein synthesis and skeletal muscle growth, chronic elevation of mTOR and/or S6K1 phosphorylation may be one of the culprits hindering muscle anabolism in obesity. Specifically, increased S6K1 signaling may act as negative regulator impairing protein synthesis in humans with obesity. Support for this notion comes from studies in animal models, and where experimentally reducing overactivated S6K1 in mice improves muscle weight and muscle fiber area, which are reduced because of obesity (Drake et al., 2010). Although impaired muscle anabolism in the latter studies could not be explained by impaired signaling through S6K1, muscle from mice with obesity were characterized by low formation of the active translation initiation eukaryotic initiation factor 4F (eIF4F; Drake et al., 2010), which signals upregulation of protein synthesis in muscle (Pain, 1996).

Relevant to the regulation of muscle protein synthesis by the formation of the active eIF4F complex in skeletal muscle is the eukaryotic translation initiation factor 4E-binding protein 1 (4E-BP1). Phosphorylation status of 4E-BP1 controls, in part, the formation of the active eIF4F complex, and where phosphorylation of 4E-BP1 enhances assembly of the eIF4F complex (Hara et al., 1998). Phosphorylation of 4E-BP1 in response to resistance exercise is lower in humans with obesity along with lower stimulation of protein synthesis in muscle of these individuals (Beals et al., 2018). Moreover, lipid oversupply to skeletal muscle, a condition that resembles the metabolic context of obesity, impairs phosphorylation of 4E-BP1 in the presence of plasma amino acids and results in lower stimulation of protein synthesis in muscle (Stephens et al., 2015). Therefore, formation of the active eIF4F complex, and in this regard the regulation of 4E-BP1 in muscle, may be key to our understanding of intracellular signaling mechanisms that impair protein synthesis in muscle of humans with obesity.

### Protein Transcription/Translation

Although heavily investigated, mechanisms regulating global protein synthesis in skeletal muscle (i.e., mTOR signaling and eIF4F complex formation) may not necessarily alone explain impaired protein metabolism that may occur in a more compartmentalized manner in muscle of humans with obesity. Therefore, investigating synthesis of proteins in muscle of humans with obesity at the level of functionally related group of proteins and/or on a protein by protein basis can provide more specific information to understand metabolism of protein in skeletal muscle in obesity. Support for this notion comes from evidence showing altered content of individual proteins in muscle of humans with obesity (Hwang et al., 2010; Kras et al., 2018), and in parallel with changes at the levels of certain transcription factors in muscle (Zapata-Bustos et al., 2021).

Reduction in type I fibers in skeletal muscle of humans with obesity (Wade et al., 1990; Hickey et al., 1995; Tanner et al., 2002; Stuart et al., 2013) can be linked specifically to lower content of the isoform of the myosin heavy chain that identifies type I fibers (i.e., MHC-I), and which is reduced in muscle of humans with obesity (Campbell et al., 2016). In this regard, non-coding RNA molecules that differentially regulate isoforms of the myosin heavy chain protein in skeletal muscle (van Rooij et al., 2009) become of particular importance for our understanding of how metabolism of protein is altered in muscle of humans with obesity. The mRNA response of the peroxisome proliferator-activated receptor-gamma coactivator (PGC)-1 α, a transcription factor upregulating mitochondrial biogenesis (Miller and Hamilton, 2012), to aerobic exercise is lower in humans with obesity (Serrano et al., 2021), possibly restricting the capacity of these individuals to upregulate protein synthesis within the mitochondrial protein pool in response to an exercise stimulus. Supporting this notion, a rat model characterized by obesity shows impaired mitochondrial protein synthesis in response to resistance exercise (Nilsson et al., 2010). Finally, upregulation of the expression of certain microRNAs in muscle of humans with obesity impairs expression of muscle mitochondrial proteins (Tran et al., 2016), as well as that of muscle insulin-like growth factor-1 (IGF-1) that regulates protein anabolism in muscle (Sullivan et al., 2020). All these lines of evidence argue for the need for investigation of mechanisms that alter protein metabolism in skeletal muscle of humans with obesity at the levels of transcription and translation of functionally related groups proteins or, most importantly, individual proteins in muscle.

### Insulin and Insulin-Like Growth Factor-1

Insulin is an anabolic hormone, and resistance to the action of insulin in skeletal muscle is a typical consequence of obesity. Lower protein synthesis in muscle of humans with obesity is observed concomitantly with lower insulin sensitivity (Guillet et al., 2009; Tran et al., 2018) and higher HbA1c values (Bak et al., 2016; Tran et al., 2018). Moreover, experimental induction of insulin resistance by intravenous infusion of lipids attenuates the increase in protein synthesis in muscle in response to plasma amino acids (Stephens et al., 2015). All these lines of evidence suggest that insulin resistance may causally be linked to impaired protein synthesis in muscle of humans with obesity. Although insulin resistance has not always been measured in the studies evaluating effects of human obesity on muscle protein turnover, it is likely that subjects with obesity in these studies have had some degree of insulin resistance. Because muscle insulin resistance differs considerably among humans with obesity (Kloting et al., 2010), differences in insulin resistance among subjects with obesity may explain discrepant finding in muscle protein synthesis in obesity reported across studies to date.

A role of insulin resistance in impairing protein metabolism in muscle of humans with obesity in a way analogous to that of glucose metabolism, however, remains to be described. In healthy subjects, insulin does not increase protein synthesis in muscle (Chow et al., 2006), but it appears to have some permissive effect with respect to stimulation of muscle protein synthesis by plasma amino acids (Abdulla et al., 2016). On the other hand, insulin does inhibit muscle protein breakdown (Chow et al., 2006; Abdulla et al., 2016). In this regard, and in the presence of resistance to the action of insulin in muscle, the expectation is that humans with obesity/insulin resistance have increased rate of muscle protein breakdown. However, humans with obesity appear to have lower muscle protein breakdown in either the fasted state (Patterson et al., 2002; Bak et al., 2016) or in the presence of hyperinsulinemia (Murton et al., 2015). Therefore, evidence to date does not describe clear role of resistance to the action of insulin in muscle in modifying protein turnover in skeletal muscle of humans with obesity.

IGF-1 controls signaling pathways in skeletal muscle largely comparable to those of insulin, and its effects are evident on both protein synthesis and breakdown (Yoshida and Delafontaine, 2020). Either acute elevation of plasma IGF-1 concentrations or chronic administration of IGF-1 increases protein synthesis in muscle of healthy subjects (Fryburg, 1994; Butterfield et al., 1997). Although plasma IGF-1 concentrations vary among individuals, including humans with obesity, overall, plasma IGF-1 concentrations are associated inversely with body mass index (Juul, 2003). No differences in muscle protein synthesis are evident between humans with obesity and lean controls in the absence of differences in plasma IGF-1 concentrations (Glynn et al., 2015; Serrano et al., 2021). However, humans with obesity that also have lower levels of plasma IGF-1 have lower muscle protein synthesis compared to lean controls (Tran et al., 2018). Moreover, expression of IGF-1 is lower in humans with obesity specifically within skeletal muscle (Sullivan et al., 2020), and which could more directly, when compared to plasma IGF-1, contribute to attenuating muscle protein synthesis in humans with obesity. Also, lower response of muscle IGF-1 expression to resistance exercise in humans with obesity (Sullivan et al., 2020) may explain blunted response of muscle protein synthesis observed in humans with obesity after resistance exercise (Beals et al., 2018). Therefore, presence of lower IGF-1 in plasma, or more importantly within skeletal muscle, in obesity may have key role in mediating lower protein synthesis in muscle of humans with obesity.

### Lipids

Lower protein synthesis in muscle in obesity is observed concurrently with increased concentrations of plasma triglycerides (Tran et al., 2018) or free fatty acids (Bak et al., 2016). Moreover, accumulation of lipids in skeletal muscle is a key feature of the metabolic state of obesity (Goodpaster et al., 2000; Sinha et al., 2002). Because lipids accumulate in muscle in response to increase in the concentrations of circulating free fatty acids (Lee et al., 2013), increase in plasma free fatty acids has been experimentally employed to investigate effects of muscle lipids on impairing protein metabolism in muscle.

Early evidence in rodents by Lang (2006) shows ~25% reduction in basal state skeletal muscle protein synthesis in response to short-term elevation in plasma lipids. This decrease in muscle protein synthesis is observed together with lower eIF4F complex formation (Lang, 2006). Formation of the eIF4F complex is also lower in muscle of rodents characterized by obesity and concomitant lipid accumulation in muscle (Drake et al., 2010). Moreover, lipid infusion in rodents completely prevents eIF4F complex formation induced by IGF-1 administration (Lang, 2006), providing evidence that lipids impair protein synthesis not only in the basal state, but also in the presence of stimuli that upregulate protein synthesis in muscle.

Initial evidence in humans showed that basal state protein synthesis in muscle, although not significantly different, was reduced by ~17% in the presence of elevated plasma free fatty acids (Katsanos et al., 2009). On the other hand, and in response to an oral amino acid anabolic stimulus, muscle protein synthesis increased normally, and despite elevated plasma free fatty acids (Katsanos et al., 2009), an effect also observed in the rodent studies discussed above (Lang, 2006). However, stimulation of muscle protein synthesis by plasma amino acids in humans is impaired by elevated plasma free fatty acids and in the presence of concomitant infusion of insulin (Stephens et al., 2015). These apparently conflicting findings between the human studies (Katsanos et al., 2009; Stephens et al., 2015) describing effects of plasma lipids on the regulation of muscle protein synthesis by amino acids are not easy to reconcile. However, are likely due to the unique experimental conditions of each study, including the plasma insulin concentrations. An ~3.5 times greater endogenous insulin response in plasma to the amino acid stimulus (Katsanos et al., 2009) may have potentially overcome impaired protein metabolism in muscle induced by the elevated plasma lipids, an effect that is less likely to occur at comparable plasma insulin concentrations (Stephens et al., 2015). Therefore, and under comparable metabolic conditions, acute increase in lipids in plasma (and muscle) appears to impair muscle protein synthesis.

Increase in skeletal muscle ceramide, a major intercellular lipid, following fat overfeeding in humans does not appear to impair the response of muscle protein synthesis to protein ingestion (Tsintzas et al., 2020). However, this evidence was obtained in overweight and obese subjects that may already present increased concentrations of ceramide in muscle. From a more mechanistic perspective, protein synthesis is reduced in myotubes in response to ceramide accumulation (Hyde et al., 2005), and lower protein synthesis under these conditions occurs together with reduced amino acid transport and intracellular amino acid availability (Hyde et al., 2005). Along these lines of evidence, rodents with obesity display lower skeletal muscle amino acid transport and uptake (Le Marchand-Brustel et al., 1982; Friedman et al., 1990). Collectively, evidence discussed in this section shows that muscle lipid accumulation may reduce protein synthesis in muscle. However, more studies are necessary to determine a causal link between muscle lipid accumulation and impaired muscle protein turnover in humans, and unravel the potential mechanisms that may sustain this response.

### Delivery of Amino Acids

Sufficient availability of amino acids in muscle is essential for protein synthesis. Availability of amino acids in muscle is regulated in part by muscle blood flow, which determines the rate of delivery of amino acids to muscle. Lower protein synthesis in muscle of subjects with obesity compared to lean subjects in the basal state is observed concurrently with lower limb blood flow in the subjects with obesity (Bak et al., 2016). Thus, reduced blood flow in muscle of humans with obesity can reduce protein synthesis secondary to reduced amino acid delivery and subsequent uptake of these amino acids by the muscle, and as seen in rodents with obesity displaying lower muscle amino acid transport and uptake (Le Marchand-Brustel et al., 1982; Friedman et al., 1990). Supporting this evidence in humans, subjects with obesity have lower concentrations of amino acids in skeletal muscle, including essential amino acids (i.e., histidine and methionine; Baker et al., 2015), which are key for regulating protein synthesis in muscle. Therefore, reductions in muscle blood flow in humans with obesity leading to reduced availability and uptake of amino acids in muscle may present a possible mechanism explaining lower protein synthesis in the muscle of humans with obesity.

Under conditions that increase blood flow to muscle, delivery and availability of amino acids to muscle also increases along with an upregulation of muscle protein synthesis (Fujita et al., 2006). The postprandial period represents a physiological circumstance that protein synthesis in muscle increases because of increase in the delivery of amino acids. Amino acid delivery to muscle increases during the postprandial period not only because of increase in plasma amino acid concentrations, but also because of an insulin-stimulated increase in muscle blood flow and muscle perfusion. Insulin increases muscle perfusion (Vincent et al., 2003), a process that increases availability of amino acids for protein synthesis in muscle (Timmerman et al., 2010). However, humans with obesity have impaired insulin-stimulated muscle perfusion (Clerk et al., 2006), which can compromise amino acid availability for protein synthesis in the muscle of these individuals.

The extent to which obesity impairs protein synthesis in muscle secondary to reduced insulin-stimulated muscle blood flow requires further investigation. However, experimental elevation in plasma lipids impairs insulin-stimulated blood flow within the muscle microvasculature (Liu et al., 2009), a process expected to also compromise delivery of plasma amino acids to muscle and, thus, muscle protein synthesis. Indeed, stimulation of muscle protein synthesis in the presence of insulin is lower under experimental conditions associated with elevated plasma lipids (Stephens et al., 2015). However, hyperinsulinemia concurrent with hyperaminoacidemia in subjects with obesity results in normal stimulation of protein synthesis in muscle (Guillet et al., 2009), and although subjects with obesity are expected to have impaired insulin-stimulated blood flow in muscle (Clerk et al., 2006). It appears that the interplay between plasma amino acid concentrations and insulin-stimulated blood flow in muscle in regulating the delivery of plasma amino acids to muscle has key role in our efforts to unravel possible defects associated with insulin-stimulated regulation of protein synthesis in muscle of humans with obesity. This is because increased delivery of amino acids to muscle results not only from increased blood flow but also increased concentrations of amino acids in plasma. Therefore, large increases in the concentrations of amino acids in plasma can mask possible impairments in insulin-stimulated blood flow in muscle that directly impair amino acid delivery and, thus, protein synthesis in muscle of humans with obesity. Clearly, there is a need for more research on how the process of amino acid delivery in obesity may impact protein synthesis in the muscle of these individuals.

## Conclusion and Future Directions

**Figure 1:**
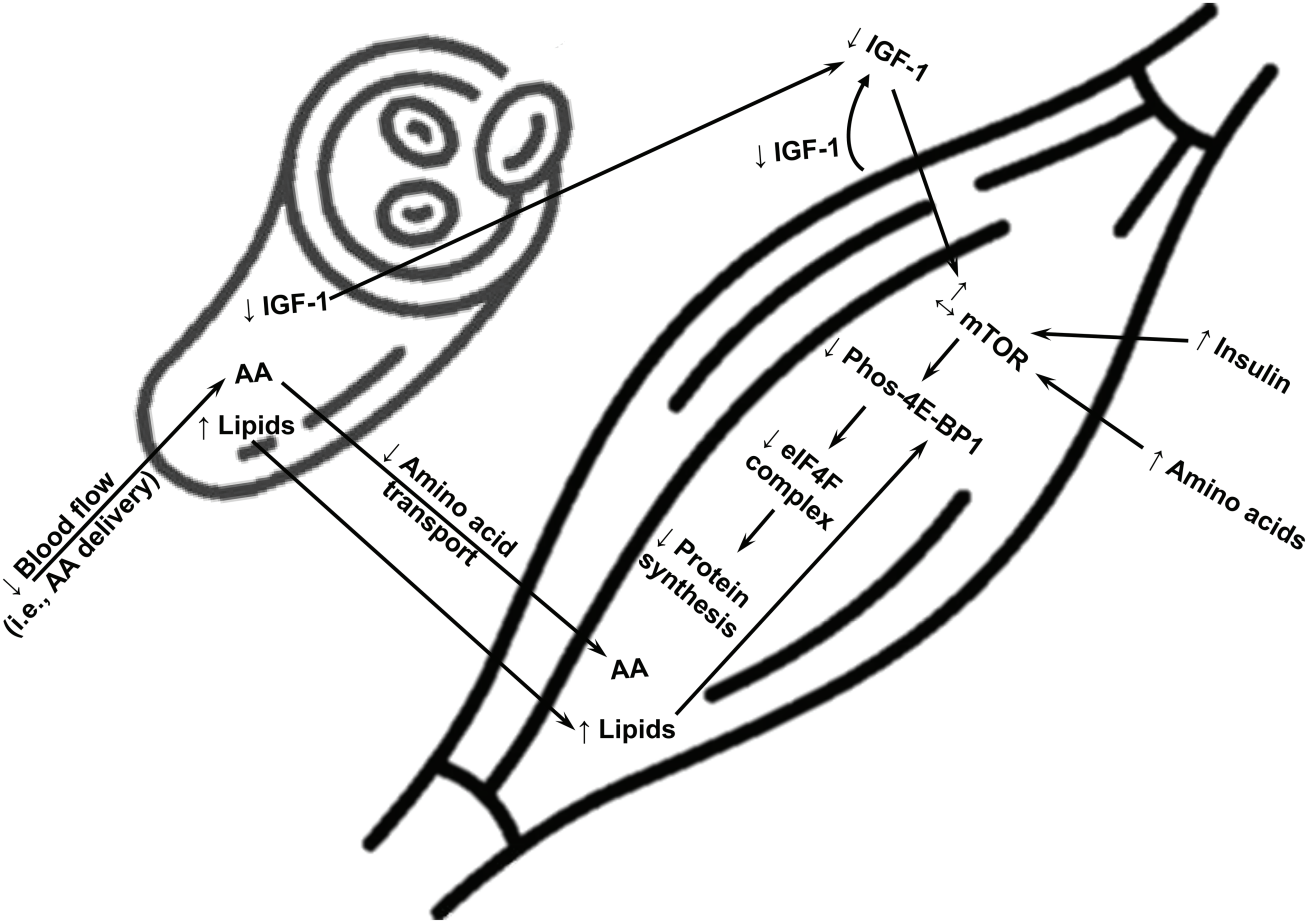
A summary of potential major mechanisms impairing synthesis of overall protein in skeletal muscle of humans with obesity. AA, amino acids; mTOR, mammalian target of rapamycin; 4E-BP1, eukaryotic translation initiation factor 4E-binding protein 1; eIF4F, translation initiation eukaryotic initiation factor 4F; IGF-1, insulin-like growth factor-1; ↑, upregulation; ↓, downregulation.

Lower protein synthesis in skeletal muscle in either the basal/fasted state and/or in response to physiological stimuli (i.e., exercise and amino acids) is a manifestation of the metabolic state of obesity, but it is not always present in humans with obesity. Because obesity is associated with heterogeneity in metabolic responses among humans (Kloting et al., 2010; Ahima and Lazar, 2013), it is only reasonable to speculate that analogous heterogeneity exists in terms of protein metabolism in skeletal muscle. Also, investigations contrasting turnover of different protein pools in muscle (i.e., myofibrillar, mitochondrial, and sarcoplasmic proteins) reveal that defects in protein turnover in humans with obesity do not occur across the entire skeletal muscle proteome. Moreover, there is time course in the development of obesity-associated impairments in muscle protein turnover, as revealed in animal models of obesity (Masgrau et al., 2012). Therefore, measurements performed in the presence of evolving vs. established obesity may contribute to the discrepant findings in the responses of protein synthesis in muscle of humans with obesity. It is likely that being on the extreme end of the spectrum of obesity-associated metabolic impairments impairs protein synthesis in muscle of humans with obesity. Current evidence supports various biological mechanisms that explain impairments in protein synthesis in skeletal muscle of humans with obesity, and which are summarized in Figure 1.

There is currently very limited data describing protein breakdown in muscle of humans with obesity. By evaluating simultaneously protein synthesis and breakdown at the individual protein level, we will be able to obtain more precise insights into defects in protein metabolism in muscle of humans with obesity. There is critical need for more studies describing intracellular signaling, transcription/translation, insulin/IGF-1 signaling, as well as the roles of plasma and muscle lipids, and amino acid delivery/blood flow in regulating protein synthesis and breakdown in muscle of humans with obesity. Future studies exploring the relative importance of different mechanisms of protein dysregulation in muscle of humans with obesity need to clearly and extensively define the metabolic characteristics of their subject populations. Also, studies need to be conducted under clearly defined, and relatively consistent, experimental conditions to allow for meaningful comparisons between study findings.

## Author Contributions

EF and CK contributed to conception of the manuscript. EF wrote the first draft of the manuscript. All authors contributed to manuscript read, revised, and approved the submitted version.

## Funding

This work was supported in part by DK123441 (CK).

## Conflict of Interest

The authors declare that the research was conducted in the absence of any commercial or financial relationships that could be construed as a potential conflict of interest.

## Publisher’s Note

All claims expressed in this article are solely those of the authors and do not necessarily represent those of their affiliated organizations, or those of the publisher, the editors and the reviewers. Any product that may be evaluated in this article, or claim that may be made by its manufacturer, is not guaranteed or endorsed by the publisher.

## References

[ref1] Abdul-Ghani M. A. Jani R. Chavez A. Molina-Carrion M. Tripathy D. Defronzo R. A. (2009). Mitochondrial reactive oxygen species generation in obese non-diabetic and type 2 diabetic participants. Diabetologia 52, 574–582. doi: 10.1007/s00125-009-1264-4, PMID: 19183935

[ref2] Abdulla H. Smith K. Atherton P. J. Idris I. (2016). Role of insulin in the regulation of human skeletal muscle protein synthesis and breakdown: a systematic review and meta-analysis. Diabetologia 59, 44–55. doi: 10.1007/s00125-015-3751-0, PMID: 26404065

[ref3] Adams S. H. (2011). Emerging perspectives on essential amino acid metabolism in obesity and the insulin-resistant state. Adv. Nutr. 2, 445–456. doi: 10.3945/an.111.000737, PMID: 22332087 PMC3226382

[ref4] Ahima R. S. Lazar M. A. (2013). Physiology. The health risk of obesity-better metrics imperative. Science 341, 856–858. doi: 10.1126/science.1241244, PMID: 23970691

[ref5] Atherton P. J. Smith K. (2012). Muscle protein synthesis in response to nutrition and exercise. J. Physiol. 590, 1049–1057. doi: 10.1113/jphysiol.2011.225003, PMID: 22289911 PMC3381813

[ref6] Ato S. Mori T. Fujita Y. Mishima T. Ogasawara R. (2021). Short-term high-fat diet induces muscle fiber type-selective anabolic resistance to resistance exercise. J. Appl. Physiol. 131, 442–453. doi: 10.1152/japplphysiol.00889.2020, PMID: 34138646

[ref7] Bak A. M. Moller A. B. Vendelbo M. H. Nielsen T. S. Viggers R. Rungby J.. (2016). Differential regulation of lipid and protein metabolism in obese vs. lean subjects before and after a 72-h fast. Am. J. Physiol. Endocrinol. Metab. 311, E224–E235. doi: 10.1152/ajpendo.00464.2015, PMID: 27245338

[ref8] Baker P. R. Boyle K. E. Koves T. R. Ilkayeva O. R. Muoio D. M. Houmard J. A.. (2015). Metabolomic analysis reveals altered skeletal muscle amino acid and fatty acid handling in obese humans. Obesity 23, 981–988. doi: 10.1002/oby.21046, PMID: 25864501 PMC4414721

[ref9] Balage M. Sinaud S. Prod’homme M. Dardevet D. Vary T. C. Kimball S. R.. (2001). Amino acids and insulin are both required to regulate assembly of the eIF4E. eIF4G complex in rat skeletal muscle. Am. J. Physiol. Endocrinol. Metab. 281, E565–E574. doi: 10.1152/ajpendo.2001.281.3.E565, PMID: 11500312

[ref10] Basisty N. Meyer J. G. Schilling B. (2018). Protein turnover in aging and longevity. Proteomics 18:e1700108. doi: 10.1002/pmic.201700108, PMID: 29453826 PMC6022828

[ref11] Beals J. W. Mackenzie R. W. A. van Vliet S. Skinner S. K. Pagni B. A. Niemiro G. M.. (2017). Protein-rich food ingestion stimulates mitochondrial protein synthesis in sedentary young adults of different BMIs. J. Clin. Endocrinol. Metab. 102, 3415–3424. doi: 10.1210/jc.2017-00360, PMID: 28911136

[ref12] Beals J. W. Skinner S. K. McKenna C. F. Poozhikunnel E. G. Farooqi S. A. van Vliet S.. (2018). Altered anabolic signalling and reduced stimulation of myofibrillar protein synthesis after feeding and resistance exercise in people with obesity. J. Physiol. 596, 5119–5133. doi: 10.1113/JP276210, PMID: 30113718 PMC6209748

[ref13] Beals J. W. Sukiennik R. A. Nallabelli J. Emmons R. S. van Vliet S. Young J. R.. (2016). Anabolic sensitivity of postprandial muscle protein synthesis to the ingestion of a protein-dense food is reduced in overweight and obese young adults. Am. J. Clin. Nutr. 104, 1014–1022. doi: 10.3945/ajcn.116.130385, PMID: 27604771

[ref14] Boden G. (2008). Obesity and free fatty acids. Endocrinol. Metab. Clin. N. Am. 37, 635–646. viii–ix. doi: 10.1016/j.ecl.2008.06.007, PMID: 18775356 PMC2596919

[ref15] Burd N. A. Tang J. E. Moore D. R. Phillips S. M. (2009). Exercise training and protein metabolism: influences of contraction, protein intake, and sex-based differences. J. Appl. Physiol. 106, 1692–1701. doi: 10.1152/japplphysiol.91351.2008, PMID: 19036897

[ref16] Butterfield G. E. Thompson J. Rennie M. J. Marcus R. Hintz R. L. Hoffman A. R. (1997). Effect of rhGH and rhIGF-I treatment on protein utilization in elderly women. Am. J. Phys. 272, E94–E99. doi: 10.1152/ajpendo.1997.272.1.E94, PMID: 9038857

[ref17] Camera D. M. Burniston J. G. Pogson M. A. Smiles W. J. Hawley J. A. (2017). Dynamic proteome profiling of individual proteins in human skeletal muscle after a high-fat diet and resistance exercise. FASEB J. 31, 5478–5494. doi: 10.1096/fj.201700531R, PMID: 28855275

[ref18] Campbell L. E. Langlais P. R. Day S. E. Coletta R. L. Benjamin T. R. De Filippis E. A.. (2016). Identification of novel changes in human skeletal muscle proteome after roux-en-Y gastric bypass surgery. Diabetes 65, 2724–2731. doi: 10.2337/db16-0004, PMID: 27207528 PMC5001187

[ref19] Carraro F. Stuart C. A. Hartl W. H. Rosenblatt J. Wolfe R. R. (1990). Effect of exercise and recovery on muscle protein synthesis in human subjects. Am. J. Phys. 259, E470–E476. doi: 10.1152/ajpendo.1990.259.4.E470 2221048

[ref20] Castets P. Lin S. Rion N. Di Fulvio S. Romanino K. Guridi M.. (2013). Sustained activation of mTORC1 in skeletal muscle inhibits constitutive and starvation-induced autophagy and causes a severe, late-onset myopathy. Cell Metab. 17, 731–744. doi: 10.1016/j.cmet.2013.03.015, PMID: 23602450

[ref21] Chevalier S. Burgos S. A. Morais J. A. Gougeon R. Bassil M. Lamarche M.. (2015). Protein and glucose metabolic responses to hyperinsulinemia, hyperglycemia, and hyperaminoacidemia in obese men. Obesity 23, 351–358. doi: 10.1002/oby.20943, PMID: 25452199

[ref22] Chomentowski P. Coen P. M. Radikova Z. Goodpaster B. H. Toledo F. G. (2011). Skeletal muscle mitochondria in insulin resistance: differences in intermyofibrillar versus subsarcolemmal subpopulations and relationship to metabolic flexibility. J. Clin. Endocrinol. Metab. 96, 494–503. doi: 10.1210/jc.2010-0822, PMID: 21106709 PMC3048328

[ref23] Chow L. S. Albright R. C. Bigelow M. L. Toffolo G. Cobelli C. Nair K. S. (2006). Mechanism of insulin’s anabolic effect on muscle: measurements of muscle protein synthesis and breakdown using aminoacyl-tRNA and other surrogate measures. Am. J. Physiol. Endocrinol. Metab. 291, E729–E736. doi: 10.1152/ajpendo.00003.2006, PMID: 16705065

[ref24] Clerk L. H. Vincent M. A. Jahn L. A. Liu Z. Lindner J. R. Barrett E. J. (2006). Obesity blunts insulin-mediated microvascular recruitment in human forearm muscle. Diabetes 55, 1436–1442. doi: 10.2337/db05-1373, PMID: 16644702

[ref25] Coen P. M. Musci R. V. Hinkley J. M. Miller B. F. (2018). Mitochondria as a target for mitigating sarcopenia. Front. Physiol. 9:1883. doi: 10.3389/fphys.2018.01883, PMID: 30687111 PMC6335344

[ref26] Denne S. C. Brechtel G. Johnson A. Liechty E. A. Baron A. D. (1995). Skeletal muscle proteolysis is reduced in noninsulin-dependent diabetes mellitus and is unaltered by euglycemic hyperinsulinemia or intensive insulin therapy. J. Clin. Endocrinol. Metab. 80, 2371–2377. doi: 10.1210/jcem.80.8.7629232 7629232

[ref27] Di Donato D. M. West D. W. Churchward-Venne T. A. Breen L. Baker S. K. Phillips S. M. (2014). Influence of aerobic exercise intensity on myofibrillar and mitochondrial protein synthesis in young men during early and late postexercise recovery. Am. J. Physiol. Endocrinol. Metab. 306, E1025–E1032. doi: 10.1152/ajpendo.00487.2013, PMID: 24595306 PMC4010655

[ref28] Donges C. E. Burd N. A. Duffield R. Smith G. C. West D. W. Short M. J.. (2012). Concurrent resistance and aerobic exercise stimulates both myofibrillar and mitochondrial protein synthesis in sedentary middle-aged men. J. Appl. Physiol. 112, 1992–2001. doi: 10.1152/japplphysiol.00166.2012, PMID: 22492939

[ref29] Drake J. C. Alway S. E. Hollander J. M. Williamson D. L. (2010). AICAR treatment for 14 days normalizes obesity-induced dysregulation of TORC1 signaling and translational capacity in fasted skeletal muscle. Am. J. Physiol. Regul. Integr. Comp. Physiol. 299, R1546–R1554. doi: 10.1152/ajpregu.00337.2010, PMID: 20844264 PMC6957371

[ref30] Durschlag R. P. Layman D. K. (1983). Skeletal muscle growth in lean and obese Zucker rats. Growth 47, 282–291. PMID: 6196256

[ref31] Falk B. Sadres E. Constantini N. Zigel L. Lidor R. Eliakim A. (2002). The association between adiposity and the response to resistance training among pre- and early-pubertal boys. J. Pediatr. Endocrinol. Metab. 15, 597–606. doi: 10.1515/JPEM.2002.15.5.597, PMID: 12014518

[ref32] Felig P. Marliss E. Cahill G. F. Jr. (1969). Plasma amino acid levels and insulin secretion in obesity. N. Engl. J. Med. 281, 811–816. doi: 10.1056/NEJM196910092811503 5809519

[ref33] Friedman J. E. Lemon P. W. Finkelstein J. A. (1990). Effect of exercise and obesity on skeletal muscle amino acid uptake. J. Appl. Physiol. 69, 1347–1352. doi: 10.1152/jappl.1990.69.4.1347, PMID: 2262452

[ref34] Fryburg D. A. (1994). Insulin-like growth factor I exerts growth hormone- and insulin-like actions on human muscle protein metabolism. Am. J. Phys. 267, E331–E336. doi: 10.1152/ajpendo.1994.267.2.E331, PMID: 8074213

[ref35] Fujita S. Rasmussen B. B. Cadenas J. G. Grady J. J. Volpi E. (2006). Effect of insulin on human skeletal muscle protein synthesis is modulated by insulin-induced changes in muscle blood flow and amino acid availability. Am. J. Physiol. Endocrinol. Metab. 291, E745–E754. doi: 10.1152/ajpendo.00271.2005, PMID: 16705054 PMC2804964

[ref36] Glynn E. L. Piner L. W. Huffman K. M. Slentz C. A. Elliot-Penry L. AbouAssi H.. (2015). Impact of combined resistance and aerobic exercise training on branched-chain amino acid turnover, glycine metabolism and insulin sensitivity in overweight humans. Diabetologia 58, 2324–2335. doi: 10.1007/s00125-015-3705-6, PMID: 26254576 PMC4793723

[ref37] Goodpaster B. H. Theriault R. Watkins S. C. Kelley D. E. (2000). Intramuscular lipid content is increased in obesity and decreased by weight loss. Metabolism 49, 467–472. doi: 10.1016/S0026-0495(00)80010-4, PMID: 10778870

[ref38] Guillet C. Delcourt I. Rance M. Giraudet C. Walrand S. Bedu M.. (2009). Changes in basal and insulin and amino acid response of whole body and skeletal muscle proteins in obese men. J. Clin. Endocrinol. Metab. 94, 3044–3050. doi: 10.1210/jc.2008-2216, PMID: 19470633

[ref39] Guillet C. Masgrau A. Boirie Y. (2011). Is protein metabolism changed with obesity? Curr. Opin. Clin. Nutr. Metab. Care 14, 89–92. doi: 10.1097/MCO.0b013e328341389e 21088567

[ref40] Hales C. M. Carroll M. D. Fryar C. D. Ogden C. L. (2020). Prevalence of obesity and severe obesity among adults: United States, 2017–2018. NCHS Data Brief 360, 1–8. 32487284

[ref41] Hara K. Yonezawa K. Weng Q. P. Kozlowski M. T. Belham C. Avruch J. (1998). Amino acid sufficiency and mTOR regulate p70 S6 kinase and eIF-4E BP1 through a common effector mechanism. J. Biol. Chem. 273, 14484–14494. PMID: 9603962 10.1074/jbc.273.23.14484

[ref42] Harber M. P. Konopka A. R. Jemiolo B. Trappe S. W. Trappe T. A. Reidy P. T. (2010). Muscle protein synthesis and gene expression during recovery from aerobic exercise in the fasted and fed states. Am. J. Physiol. Regul. Integr. Comp. Physiol. 299, R1254–R1262. doi: 10.1152/ajpregu.00348.2010, PMID: 20720176

[ref43] Herrero M. C. Remesar X. Blade C. Arola L. (1997). Muscle amino acid pattern in obese rats. Int. J. Obes. Relat. Metab. Disord. 21, 698–703. doi: 10.1038/sj.ijo.0800464, PMID: 15481771

[ref44] Hickey M. S. Carey J. O. Azevedo J. L. Houmard J. A. Pories W. J. Israel R. G.. (1995). Skeletal muscle fiber composition is related to adiposity and in vitro glucose transport rate in humans. Am. J. Phys. 268, E453–E457. doi: 10.1152/ajpendo.1995.268.3.E453, PMID: 7900793

[ref45] Hojlund K. Yi Z. Lefort N. Langlais P. Bowen B. Levin K.. (2010). Human ATP synthase beta is phosphorylated at multiple sites and shows abnormal phosphorylation at specific sites in insulin-resistant muscle. Diabetologia 53, 541–551. doi: 10.1007/s00125-009-1624-0, PMID: 20012595

[ref46] Hulston C. J. Woods R. M. Dewhurst-Trigg R. Parry S. A. Gagnon S. Baker L.. (2018). Resistance exercise stimulates mixed muscle protein synthesis in lean and obese young adults. Physiol. Rep. 6:e13799. doi: 10.14814/phy2.13799, PMID: 30009507 PMC6046643

[ref47] Hwang H. Bowen B. P. Lefort N. Flynn C. R. De Filippis E. A. Roberts C.. (2010). Proteomics analysis of human skeletal muscle reveals novel abnormalities in obesity and type 2 diabetes. Diabetes 59, 33–42. doi: 10.2337/db09-0214, PMID: 19833877 PMC2797941

[ref48] Hyde R. Hajduch E. Powell D. J. Taylor P. M. Hundal H. S. (2005). Ceramide down-regulates system A amino acid transport and protein synthesis in rat skeletal muscle cells. FASEB J. 19, 461–463. doi: 10.1096/fj.04-2284fje, PMID: 15611152

[ref49] Jaleel A. Henderson G. C. Madden B. J. Klaus K. A. Morse D. M. Gopala S.. (2010). Identification of de novo synthesized and relatively older proteins: accelerated oxidative damage to de novo synthesized apolipoprotein A-1 in type 1 diabetes. Diabetes 59, 2366–2374. doi: 10.2337/db10-0371, PMID: 20622162 PMC3279529

[ref50] Jaleel A. Short K. R. Asmann Y. W. Klaus K. A. Morse D. M. Ford G. C.. (2008). In vivo measurement of synthesis rate of individual skeletal muscle mitochondrial proteins. Am. J. Physiol. Endocrinol. Metab. 295, E1255–E1268. doi: 10.1152/ajpendo.90586.2008, PMID: 18765679 PMC2584812

[ref51] Juul A. (2003). Serum levels of insulin-like growth factor I and its binding proteins in health and disease. Growth Hormon. IGF Res. 13, 113–170. doi: 10.1016/S1096-6374(03)00038-8, PMID: 12914749

[ref52] Katsanos C. S. Aarsland A. Cree M. G. Wolfe R. R. (2009). Muscle protein synthesis and balance responsiveness to essential amino acids ingestion in the presence of elevated plasma free fatty acid concentrations. J. Clin. Endocrinol. Metab. 94, 2984–2990. doi: 10.1210/jc.2008-2686, PMID: 19454587 PMC2730875

[ref53] Katsanos C. S. Mandarino L. J. (2011). Protein metabolism in human obesity: a shift in focus from whole-body to skeletal muscle. Obesity 19, 469–475. doi: 10.1038/oby.2010.290, PMID: 21164506

[ref54] Khamzina L. Veilleux A. Bergeron S. Marette A. (2005). Increased activation of the mammalian target of rapamycin pathway in liver and skeletal muscle of obese rats: possible involvement in obesity-linked insulin resistance. Endocrinology 146, 1473–1481. doi: 10.1210/en.2004-0921, PMID: 15604215

[ref55] Kloting N. Fasshauer M. Dietrich A. Kovacs P. Schon M. R. Kern M.. (2010). Insulin-sensitive obesity. Am. J. Physiol. Endocrinol. Metab. 299, E506–E515. doi: 10.1152/ajpendo.00586.2009 20570822

[ref56] Kobayashi H. Borsheim E. Anthony T. G. Traber D. L. Badalamenti J. Kimball S. R.. (2003). Reduced amino acid availability inhibits muscle protein synthesis and decreases activity of initiation factor eIF2B. Am. J. Physiol. Endocrinol. Metab. 284, E488–E498. doi: 10.1152/ajpendo.00094.2002, PMID: 12556349

[ref57] Kouw I. W. K. van Dijk J. W. Horstman A. M. H. Kramer I. F. Goessens J. P. B. van Dielen F. M. H.. (2019). Basal and postprandial myofibrillar protein synthesis rates do not differ between lean and obese middle-aged men. J. Nutr. 149, 1533–1542. doi: 10.1093/jn/nxz104, PMID: 31174213 PMC6736155

[ref58] Kras K. A. Langlais P. R. Hoffman N. Roust L. R. Benjamin T. R. De Filippis E. A.. (2018). Obesity modifies the stoichiometry of mitochondrial proteins in a way that is distinct to the subcellular localization of the mitochondria in skeletal muscle. Metabolism 89, 18–26. doi: 10.1016/j.metabol.2018.09.006, PMID: 30253140 PMC6221946

[ref59] Kruse R. Vind B. F. Petersson S. J. Kristensen J. M. Hojlund K. (2015). Markers of autophagy are adapted to hyperglycaemia in skeletal muscle in type 2 diabetes. Diabetologia 58, 2087–2095. doi: 10.1007/s00125-015-3654-0, PMID: 26048236

[ref60] Lang C. H. (2006). Elevated plasma free fatty acids decrease basal protein synthesis, but not the anabolic effect of leucine, in skeletal muscle. Am. J. Physiol. Endocrinol. Metab. 291, E666–E674. doi: 10.1152/ajpendo.00065.2006 16684854

[ref61] Lanza-Jacoby S. Kaplan M. L. (1984). Alterations in skeletal muscle proteins in obese and nonobese rats. Int. J. Obes. 8, 451–456. PMID: 6519905

[ref62] Le Marchand-Brustel Y. Moutard N. Freychet P. (1982). Aminoisobutyric acid transport in soleus muscles of lean and gold thioglucose-obese mice. Am. J. Phys. 243, E74–E79. PMID: 6807103 10.1152/ajpendo.1982.243.1.E74

[ref63] Lee S. Boesch C. Kuk J. L. Arslanian S. (2013). Effects of an overnight intravenous lipid infusion on intramyocellular lipid content and insulin sensitivity in African-American versus Caucasian adolescents. Metabolism 62, 417–423. doi: 10.1016/j.metabol.2012.09.007, PMID: 23122836 PMC3574210

[ref64] Lefort N. Glancy B. Bowen B. Willis W. T. Bailowitz Z. De Filippis E. A.. (2010). Increased reactive oxygen species production and lower abundance of complex I subunits and carnitine palmitoyltransferase 1B protein despite normal mitochondrial respiration in insulin-resistant human skeletal muscle. Diabetes 59, 2444–2452. doi: 10.2337/db10-0174, PMID: 20682693 PMC3279558

[ref65] Lennmarken C. Sandstedt S. von Schenck H. Larsson J. (1986). Skeletal muscle function and metabolism in obese women. JPEN J. Parenter. Enteral Nutr. 10, 583–587. doi: 10.1177/0148607186010006583 3540333

[ref66] Liu Z. Jahn L. A. Wei L. Long W. Barrett E. J. (2002). Amino acids stimulate translation initiation and protein synthesis through an Akt-independent pathway in human skeletal muscle. J. Clin. Endocrinol. Metab. 87, 5553–5558. doi: 10.1210/jc.2002-020424, PMID: 12466352

[ref67] Liu Z. Liu J. Jahn L. A. Fowler D. E. Barrett E. J. (2009). Infusing lipid raises plasma free fatty acids and induces insulin resistance in muscle microvasculature. J. Clin. Endocrinol. Metab. 94, 3543–3549. doi: 10.1210/jc.2009-0027, PMID: 19567533 PMC2741712

[ref68] Masgrau A. Mishellany-Dutour A. Murakami H. Beaufrere A. M. Walrand S. Giraudet C.. (2012). Time-course changes of muscle protein synthesis associated with obesity-induced lipotoxicity. J. Physiol. 590, 5199–5210. doi: 10.1113/jphysiol.2012.238576, PMID: 22802586 PMC3497572

[ref69] Miller B. F. (2007). Human muscle protein synthesis after physical activity and feeding. Exerc. Sport Sci. Rev. 35, 50–55. doi: 10.1097/jes.0b013e31803eac78 17417050

[ref70] Miller B. F. Hamilton K. L. (2012). A perspective on the determination of mitochondrial biogenesis. Am. J. Physiol. Endocrinol. Metab. 302, E496–E499. doi: 10.1152/ajpendo.00578.2011, PMID: 22205627 PMC3311289

[ref71] Moller A. B. Kampmann U. Hedegaard J. Thorsen K. Nordentoft I. Vendelbo M. H.. (2017). Altered gene expression and repressed markers of autophagy in skeletal muscle of insulin resistant patients with type 2 diabetes. Sci. Rep. 7:43775. doi: 10.1038/srep43775, PMID: 28252104 PMC5333153

[ref72] Murton A. J. Marimuthu K. Mallinson J. E. Selby A. L. Smith K. Rennie M. J.. (2015). Obesity appears to be associated With altered muscle protein synthetic and breakdown responses to increased nutrient delivery in older men, but not reduced muscle mass or contractile function. Diabetes 64, 3160–3171. doi: 10.2337/db15-0021 26015550

[ref73] Nilsson M. I. Greene N. P. Dobson J. P. Wiggs M. P. Gasier H. G. Macias B. R.. (2010). Insulin resistance syndrome blunts the mitochondrial anabolic response following resistance exercise. Am. J. Physiol. Endocrinol. Metab. 299, E466–E474. doi: 10.1152/ajpendo.00118.2010, PMID: 20606077

[ref74] Pain V. M. (1996). Initiation of protein synthesis in eukaryotic cells. Eur. J. Biochem. 236, 747–771. doi: 10.1111/j.1432-1033.1996.00747.x 8665893

[ref75] Patterson B. W. Horowitz J. F. Wu G. Watford M. Coppack S. W. Klein S. (2002). Regional muscle and adipose tissue amino acid metabolism in lean and obese women. Am. J. Physiol. Endocrinol. Metab. 282, E931–E936. doi: 10.1152/ajpendo.00359.2001, PMID: 11882515

[ref76] Petersen L. Schnohr P. Sorensen T. I. (2004). Longitudinal study of the long-term relation between physical activity and obesity in adults. Int. J. Obes. Relat. Metab. Disord. 28, 105–112. doi: 10.1038/sj.ijo.0802548 14647181

[ref77] Pileggi C. A. Parmar G. Harper M. E. (2021). The lifecycle of skeletal muscle mitochondria in obesity. Obes. Rev. 22:e13164. doi: 10.1111/obr.13164, PMID: 33442950

[ref78] Santra M. Dill K. A. de Graff A. M. R. (2019). Proteostasis collapse is a driver of cell aging and death. Proc. Natl. Acad. Sci. U. S. A. 116, 22173–22178. doi: 10.1073/pnas.1906592116, PMID: 31619571 PMC6825304

[ref79] Serrano N. Tran L. Hoffman N. Roust L. De Filippis E. A. Carroll C. C.. (2021). Lack of increase in muscle mitochondrial protein synthesis During the course of aerobic exercise and its recovery in the fasting state irrespective of obesity. Front. Physiol. 12:702742. doi: 10.3389/fphys.2021.702742, PMID: 34408662 PMC8365357

[ref80] Sinha R. Dufour S. Petersen K. F. LeBon V. Enoksson S. Ma Y. Z.. (2002). Assessment of skeletal muscle triglyceride content by (1)H nuclear magnetic resonance spectroscopy in lean and obese adolescents: relationships to insulin sensitivity, total body fat, and central adiposity. Diabetes 51, 1022–1027. doi: 10.2337/diabetes.51.4.1022, PMID: 11916921

[ref81] Sitnick M. Bodine S. C. Rutledge J. C. (2009). Chronic high fat feeding attenuates load-induced hypertrophy in mice. J. Physiol. 587, 5753–5765. doi: 10.1113/jphysiol.2009.180174, PMID: 19822547 PMC2805383

[ref82] Smeuninx B. McKendry J. Wilson D. Martin U. Breen L. (2017). Age-related anabolic resistance of myofibrillar protein synthesis is exacerbated in obese inactive individuals. J. Clin. Endocrinol. Metab. 102, 3535–3545. doi: 10.1210/jc.2017-00869, PMID: 28911148 PMC5587073

[ref83] Smiles W. J. Churchward-Venne T. A. van Loon L. J. C. Hawley J. A. Camera D. M. (2019). A single bout of strenuous exercise overcomes lipid-induced anabolic resistance to protein ingestion in overweight, middle-aged men. FASEB J. 33, 7009–7017. doi: 10.1096/fj.201801917R, PMID: 30840513

[ref84] Stenholm S. Harris T. B. Rantanen T. Visser M. Kritchevsky S. B. Ferrucci L. (2008). Sarcopenic obesity: definition, cause and consequences. Curr. Opin. Clin. Nutr. Metab. Care 11, 693–700. doi: 10.1097/MCO.0b013e328312c37d, PMID: 18827572 PMC2633408

[ref85] Stephens F. B. Chee C. Wall B. T. Murton A. J. Shannon C. E. van Loon L. J.. (2015). Lipid-induced insulin resistance is associated with an impaired skeletal muscle protein synthetic response to amino acid ingestion in healthy young men. Diabetes 64, 1615–1620. doi: 10.2337/db14-0961, PMID: 25524913

[ref86] Struder H. K. Hollmann W. Platen P. Wostmann R. Weicker H. Molderings G. J. (1999). Effect of acute and chronic exercise on plasma amino acids and prolactin concentrations and on [3H]ketanserin binding to serotonin2A receptors on human platelets. Eur. J. Appl. Physiol. Occup. Physiol. 79, 318–324. doi: 10.1007/s004210050514, PMID: 10090630

[ref87] Stuart C. A. McCurry M. P. Marino A. South M. A. Howell M. E. Layne A. S.. (2013). Slow-twitch fiber proportion in skeletal muscle correlates with insulin responsiveness. J. Clin. Endocrinol. Metab. 98, 2027–2036. doi: 10.1210/jc.2012-3876, PMID: 23515448 PMC3644602

[ref88] Stump C. S. Henriksen E. J. Wei Y. Sowers J. R. (2006). The metabolic syndrome: role of skeletal muscle metabolism. Ann. Med. 38, 389–402. doi: 10.1080/07853890600888413 17008303

[ref89] Sullivan B. P. Weiss J. A. Nie Y. Garner R. T. Drohan C. J. Kuang S.. (2020). Skeletal muscle IGF-1 is lower at rest and after resistance exercise in humans with obesity. Eur. J. Appl. Physiol. 120, 2835–2846. doi: 10.1007/s00421-020-04509-z, PMID: 32989478

[ref90] Tanner C. J. Barakat H. A. Dohm G. L. Pories W. J. MacDonald K. G. Cunningham P. R.. (2002). Muscle fiber type is associated with obesity and weight loss. Am. J. Physiol. Endocrinol. Metab. 282, E1191–E1196. doi: 10.1152/ajpendo.00416.2001, PMID: 12006347

[ref91] Timmerman K. L. Lee J. L. Fujita S. Dhanani S. Dreyer H. C. Fry C. S.. (2010). Pharmacological vasodilation improves insulin-stimulated muscle protein anabolism but not glucose utilization in older adults. Diabetes 59, 2764–2771. doi: 10.2337/db10-0415, PMID: 20724580 PMC2963534

[ref92] Tipton K. D. Hamilton D. L. Gallagher I. J. (2018). Assessing the role of muscle protein breakdown in response to nutrition and exercise in humans. Sports Med. 48, 53–64. doi: 10.1007/s40279-017-0845-5, PMID: 29368185 PMC5790854

[ref93] Tomlinson D. J. Erskine R. M. Morse C. I. Winwood K. Onambele-Pearson G. (2016). The impact of obesity on skeletal muscle strength and structure through adolescence to old age. Biogerontology 17, 467–483. doi: 10.1007/s10522-015-9626-4, PMID: 26667010 PMC4889641

[ref94] Tran L. Hanavan P. D. Campbell L. E. De Filippis E. Lake D. F. Coletta D. K.. (2016). Prolonged exposure of primary human muscle cells to plasma fatty acids associated with obese phenotype induces persistent suppression of muscle mitochondrial ATP synthase beta subunit. PLoS One 11:e0160057. doi: 10.1371/journal.pone.0160057, PMID: 27532680 PMC4988792

[ref95] Tran L. Kras K. A. Hoffman N. Ravichandran J. Dickinson J. M. D’Lugos A.. (2018). Lower fasted-state but greater increase in muscle protein synthesis in response to elevated plasma amino acids in obesity. Obesity 26, 1179–1187. doi: 10.1002/oby.22213, PMID: 29896930 PMC6078204

[ref96] Tran L. Langlais P. R. Hoffman N. Roust L. Katsanos C. S. (2019). Mitochondrial ATP synthase beta-subunit production rate and ATP synthase specific activity are reduced in skeletal muscle of humans with obesity. Exp. Physiol. 104, 126–135. doi: 10.1113/EP087278, PMID: 30362197 PMC6312454

[ref97] Tremblay F. Krebs M. Dombrowski L. Brehm A. Bernroider E. Roth E.. (2005). Overactivation of S6 kinase 1 as a cause of human insulin resistance during increased amino acid availability. Diabetes 54, 2674–2684. doi: 10.2337/diabetes.54.9.2674, PMID: 16123357

[ref98] Tremblay F. Marette A. (2001). Amino acid and insulin signaling via the mTOR/p70 S6 kinase pathway. A negative feedback mechanism leading to insulin resistance in skeletal muscle cells. J. Biol. Chem. 276, 38052–38060. doi: 10.1074/jbc.M106703200, PMID: 11498541

[ref99] Tsintzas K. Jones R. Pabla P. Mallinson J. Barrett D. A. Kim D. H.. (2020). Effect of acute and short-term dietary fat ingestion on postprandial skeletal muscle protein synthesis rates in middle-aged, overweight and obese men. Am. J. Physiol. Endocrinol. Metab. 318, E417–E429. doi: 10.1152/ajpendo.00344.2019, PMID: 31910028

[ref100] Valenzuela P. L. Maffiuletti N. A. Tringali G. De Col A. Sartorio A. (2020). Obesity-associated poor muscle quality: prevalence and association with age, sex, and body mass index. BMC Musculoskelet. Disord. 21:200. doi: 10.1186/s12891-020-03228-y, PMID: 32234006 PMC7110672

[ref101] van Rooij E. Quiat D. Johnson B. A. Sutherland L. B. Qi X. Richardson J. A.. (2009). A family of microRNAs encoded by myosin genes governs myosin expression and muscle performance. Dev. Cell 17, 662–673. doi: 10.1016/j.devcel.2009.10.013, PMID: 19922871 PMC2796371

[ref102] Vincent M. A. Barrett E. J. Lindner J. R. Clark M. G. Rattigan S. (2003). Inhibiting NOS blocks microvascular recruitment and blunts muscle glucose uptake in response to insulin. Am. J. Physiol. Endocrinol. Metab. 285, E123–E129. doi: 10.1152/ajpendo.00021.2003, PMID: 12791603

[ref103] Wade A. J. Marbut M. M. Round J. M. (1990). Muscle fibre type and aetiology of obesity. Lancet 335, 805–808. doi: 10.1016/0140-6736(90)90933-V, PMID: 1969558

[ref104] Wilkinson S. B. Phillips S. M. Atherton P. J. Patel R. Yarasheski K. E. Tarnopolsky M. A.. (2008). Differential effects of resistance and endurance exercise in the fed state on signalling molecule phosphorylation and protein synthesis in human muscle. J. Physiol. 586, 3701–3717. doi: 10.1113/jphysiol.2008.153916, PMID: 18556367 PMC2538832

[ref105] Wolfe R. R. (2002). Regulation of muscle protein by amino acids. J. Nutr. 132, 3219S–3224S. doi: 10.1093/jn/131.10.3219S 12368421

[ref106] Ye R. Onodera T. Scherer P. E. (2019). Lipotoxicity and beta cell maintenance in obesity and type 2 diabetes. J. Endocr. Soc. 3, 617–631. doi: 10.1210/js.2018-00372, PMID: 30834357 PMC6391718

[ref107] Yoshida T. Delafontaine P. (2020). Mechanisms of IGF-1-mediated regulation of skeletal muscle hypertrophy and atrophy. Cell 9, 1–25. doi: 10.3390/cells9091970, PMID: 32858949 PMC7564605

[ref108] Zapata-Bustos R. Finlayson J. Langlais P. R. Coletta D. K. Luo M. Grandjean D.. (2021). Altered transcription factor expression responses to exercise in insulin resistance. Front. Physiol. 12:649461. doi: 10.3389/fphys.2021.649461, PMID: 33897458 PMC8058368

